# Synthesis of mucin-type *O*-glycan probes as aminopropyl glycosides

**DOI:** 10.3762/bjoc.9.218

**Published:** 2013-09-13

**Authors:** David Benito-Alifonso, Rachel A Jones, Anh-Tuan Tran, Hannah Woodward, Nichola Smith, M Carmen Galan

**Affiliations:** 1 School of Chemistry, University of Bristol, Cantock’s Close, Bristol BS8 1TS, UK Fax: (0)1179298611, Tel: (0)1179287654; 2 Novartis Institute for Biomolecular Research, Novartis Horsham Research Centre, Horsham RH12 5AB, UK

**Keywords:** glycosylation, mucin-type oligosaccharides, *O*-glycans, oligosaccharide synthesis

## Abstract

The chemical synthesis of a series of mucin-type oligosaccharide fragments **1**–**7** containing an α-linked aminopropyl spacer ready for glycoarray attachment is reported. A highly convergent and stereoselective strategy that employs two different orthogonal protected galactosamine building blocks was used to access all of the targets. A tandem deprotection sequence, that did not require chromatography-based purification between steps, was employed to globally unmask all protecting groups and all final targets were isolated in good to excellent yields.

## Introduction

Mucins are heavily glycosylated glycoproteins that may be membrane-associated or secreted in gel form and play an important biological role in the respiratory and intestinal tracks [1–3]. Mucins create a plethora of potential binding sites for commensal and pathogenic microbes, and are also ligands for the targeting of leucocytes to endothelial cells. Secreted mucins found in the intestinal mucus gel and the glycocalyx contain hundreds of different mucin type, *O*-linked oligosaccharides and though is widely accepted that mucins form a protective layer over the epithelium, the mechanisms to achieve this are poorly understood [4–5].

A characteristic feature of all mucins is the linking region of the protein backbone with the saccharide side-chain, which is typically an α-linked 2-acetamido-2-deoxy-D-galactose to serine or threonine. This saccharide forms the inner part of the characteristic core oligosaccharides from where glycans are extended through common core (di- and trisaccharide) structures, cores 1–8 [6–8]. The mucin-type oligosaccharides identified to date have remarkable structural diversity and present a significant challenge for synthesis [7–8].

The efficient preparation of structurally defined oligosaccharide fragments of this class, in sufficient purity and amounts and ready to be immobilized into carbohydrate arrays for high-throughput biological analysis is crucial to help us understand the biological and disease implications of these glycosylation patterns at both molecular and functional levels. However, despite the need for functional studies of *O*-glycosylation patterns, there are comparatively few reported syntheses leading to the basic mucin-type cores [9–14] than there are available for other types of glycosides and even fewer that lead to fragments ready to be conjugated to glycoarray platforms [15]. One of the most troublesome steps in previous approaches is the synthesis of the α-glycosidic linkage between 2-acetamido-2-deoxy-D-galactopyranose monosaccharide and the side-chain hydroxy groups.

**Figure 1:**
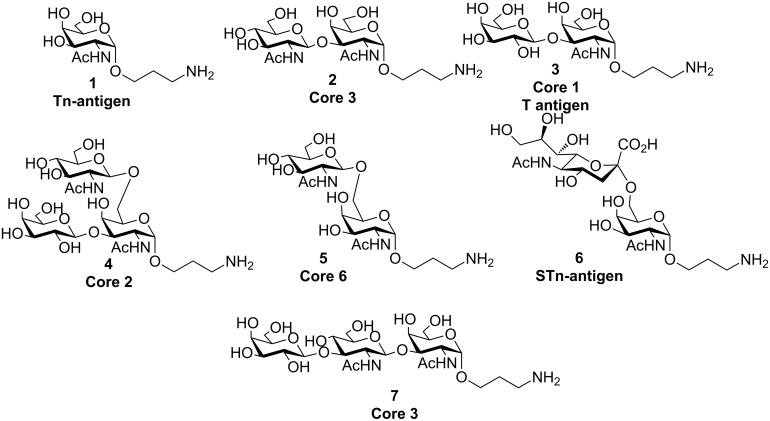
Structure of mucin-type oligosaccharide fragments synthesized.

Herein we report a convergent approach for the highly efficient synthesis of mucin type *O*-glycan fragments **1**–**7** (Figure 1) containing an α-linked 3-aminopropyl-spacer ready for covalent attachment to glycoarray platforms.

## Results and Discussion

**Scheme 1:**
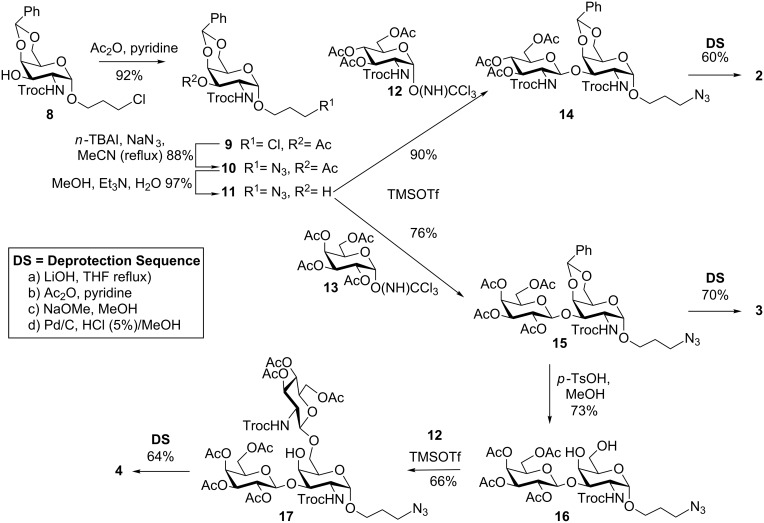
Synthesis of glycan targets **2**–**4**.

A versatile blockwise strategy was employed to prepare targets **2**–**4**, whereby each glycosylation product yielded compounds that were either deprotected to give the final products or became the acceptor for the next glycosylation step. The synthesis started from orthogonally protected 3-chloropropyl-α-linked galactosamine derivative **8** as the key intermediate. This was prepared in a three-step, high yielding stereoselective synthesis from commercially available galactosamine hydrochloride in 66% yield as previously reported [16]. From chloride **8**, 3-azidopropyl moiety **11** was accessed in a three-step process in 79% overall yield. The sequence involved temporary protection of the free hydroxy at C-3 by reaction with acetic anhydride and pyridine, S_N_2 displacement of the chloride in monosaccharide **9** with *n*-tetrabutylammonium iodide (*n*–TBAI) in refluxing acetonitrile followed by the addition of NaN_3_. Ester removal was achieved in a mixture of Et_3_N, methanol and water (Scheme 1). Attempts to obtain azide **11** directly from chloride **9** by halide displacement with NaI and NaN_3_ gave unreliable results and produced inseparable mixtures of **11** and side products. Regio- and stereoselective glycosylation of glycosyl acceptor **11** was achieved with trichloroacetimidate donors **12** [17] or **13** [18], which bear C-2 protecting groups *N*-trichloroethylcarbamate (*N*-Troc) and acetate (OAc), respectively, and which are able to undergo neighbouring group participation, to ensure β-anomeric selectivity in the glycosylation reaction [19–20]. Core 1 and core 3 disaccharide precursors **14** and **15** were then prepared in the presence of catalytic TMSOTf in respective yields of 90% and 76%. Global deprotection of the assembled oligomers was achieved by a tandem process that required no purifications between steps, as the reactions proceeded cleanly as shown by TLC. Thus, the acetamido groups in the oligosaccharides were introduced by first removing the *N*-Troc protecting group using LiOH in THF at reflux, followed by complete acetylation in pyridine and acetic anhydride. It is important to highlight that the use of activated Zn dust in acetic acid to reductively convert the *N*-Troc-protecting group to the corresponding amine, resulted in partial reduction of the azide group in the linker. We found that deacylation with LiOH gave better yields, cleaner reaction mixtures and moreover the conditions are compatible with other functionalities that are susceptible to reduction, such as azides. After aqueous workup, the crude materials were subjected to removal of the acetyl groups using sodium hydroxide in methanol (pH = 11) followed by concomitant acetal hydrolysis and azide reduction using hydrogen and palladium on charcoal (Pd/C) in methanolic HCl (5%) to yield final targets **2** and **3** in 60% and 70% yield, respectively (Scheme 1). Access to branched core 2 trisaccharide **4** was accomplished by 4,6-*O*-benzylidene acetal cleavage from disaccharide **15** using a modified procedure from Chang et al. [21] whereby reaction with *p*-TsOH in MeOH under sonication yielded 4,6-diol **16** in less than 30 minutes at room temperature. Disaccharide **16** was then regio- and stereoselectively glycosylated with *N*-Troc protected glucosamine donor **12** taking advantage of the higher reactivity of the primary OH at C-6 with respect to the C-4 OH group and trisaccharide **17** was obtained in 66% yield (Scheme 1). The same deprotection sequence as previously described for targets **2** and **3** was employed and unprotected trisaccharide **4** was isolated in 64% yield.

For an expedient synthesis of disaccharides **5** and **6**, which are extended at C-6 of the galactosamine moiety with either a β-linked *N*-acetylglucosamine or α-linked Neu5Ac, 3,4-*O*-isopropylidene protection of α-linked azido-propyl *N*-acetyl galactosamine derivative **18** [22] was carried out in DMF using *p*-TsOH and 2,2-dimethoxypropane to afford glycosyl acceptor **19** in 77% yield as the only regioisomer ready for glycosylation. To access disaccharide **20b**, trichloroacetimidate glycoside donor **12** was reacted with **19** in the presence of catalytic TMSOTf to yield an inseparable mixture of disaccharide product **20a** and unreacted starting material. Acetal cleavage with *p*-TsOH in MeOH under sonication followed by acetylation of the free OH groups using Ac_2_O and pyridine afforded the separation of the components and disaccharide **20b** was then isolated with complete stereocontrol in 41% yield after the 3 steps. Adamantanyl thiosialosides have been shown to have high reactivity under NIS/TfOH promotion conditions in nitrile solvents at −35 °C and higher α-selectivities than other sialosyl donors [23]. Under our reaction conditions, sialylated disaccharide **22a** was prepared as a 3/1 (α/β) mixture using 1-thioadamantyl sialoside **21** as the glycoside donor upon reaction with **19**, using NIS in combination with catalytic TMSOTf to activate the thioglycoside donor. The NMR data of **22a** unambiguously confirmed the presence of α-sialyl linkage (Δδ [H-9’a–H-9’b] = 0.62 ppm and *J*_7’,8’_ = 4.8 Hz) [24]. Acetal deprotection was subsequently carried out using *p*-TsOH in MeOH and the α-anomer of disaccharide **22b** was isolated by silica gel column chromatography in 40% yield after the two steps.

**Scheme 2:**
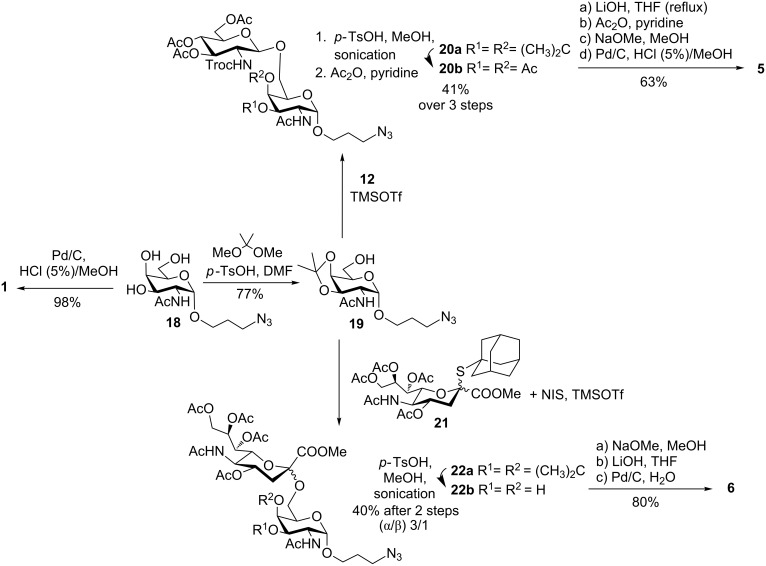
Synthesis of disaccharide targets **5** and **6**.

Deprotection of disaccharide **20b** was carried out using the same sequential approach previously described for the neutral targets **2**–**4** and compound **5** was isolated in 63% yield after the 4-step deprotection sequence. The tandem deprotection sequence was also amenable to sialylated **22b**, as the ester groups were removed by reaction with sodium methoxide in methanol followed by saponification of the methyl ester with LiOH at room temperature and subsequent azide reduction catalysed by hydrogen and Pd/C in water to give target **6** in 80% overall yield (Scheme 2). The presence of the α-sialyl linkage in **6** was also unambiguously confirmed by the NMR data (H-3eq’ δ = 2.25 ppm, Δδ [H-9’a–H-9’b] = 0.36 ppm) [24]. Then, carbohydrate antigen **1** was obtained also by catalytic hydrogenolysis with Pd/C in methanolic HCl of **18** to yield the amine-containing derivative in 98% yield.

**Scheme 3:**
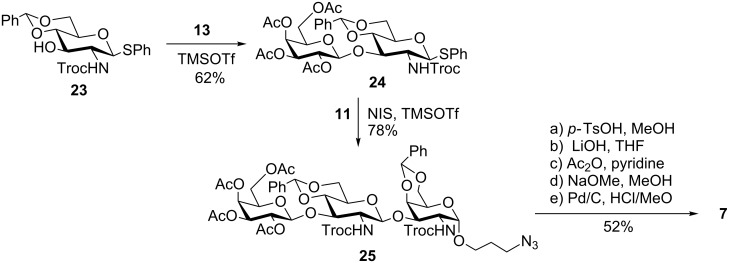
Orthogonal approach into core 3 target **7**.

To access core 3 linear trisaccharide **7**, we decided to employ an orthogonal, chemo- and stereoselective glycosylation approach [25–26], which unlike traditional strategies for oligosaccharide assembly, are based on the ability to control the reactivity of anomeric leaving groups by using carbohydrate building blocks containing leaving groups with orthogonal reactivities that can be activated independently. Saccharide building blocks can then be assembled without the need for additional protecting-group manipulations between glycosylations and the coupling sequence can be performed with glycosyl donors and acceptors that both have a free hydroxy group [27]. In this report, we employed the difference in reactivity of trichloroacetimidates and thioglycoside donors. In general, trichloroacetimidates are activated by strong Lewis acids such as TMSOTf [28], while the more stable thioglycosides require the presence of a more electrophilic species such as *N-*iodosuccinimide/TMSOTf combinations [29–30]. To that end, thioglycoside building block **23** bearing a free OH at C-3 was synthesized following reported procedures [16] and subjected to a chemo- and stereoselective glycosylation reaction with peracetylated trichloroacetimidate donor **13** in the presence of catalytic TMSOTf. Disaccharide **24** was obtained in 62% yield and without any further protecting-group manipulations, the thioglycoside disaccharide was subsequently activated as a glycosyl donor using NIS/TMSOTf and coupled to 4,6-*O*-benzylidene-protected glycosyl acceptor **11** to afford trisaccharide **25** in 78% yield (Scheme 3). With the trisaccharide in hand, we proceeded to globally deprotect the assembled oligomer using a similar tandem reaction sequence as before that involved no column chromatography in between steps. In brief, selective cleavage of the 4,6-*O*-benzylidene acetals using *p*-TsOH in MeOH under sonication followed by removal of the *N*-Troc groups in the presence of LiOH and subsequent acetylation with pyridine and acetic anhydride formed the desired acetamido functionalities, removal of the ester groups under basic conditions was followed by reduction of the azide group in the linker by catalytic hydrogenolysis using hydrogen and Pd/C to give trisaccharide **7** in 52% yield, after the 5 step unmasking sequence.

## Conclusion

A highly convergent and stereoselective strategy has been used to access mucin-type glycan targets **1**–**7**. The approach employs orthogonal protected galactosamine building blocks **11** and **18** already containing an α-linked azidopropyl spacer as the starting versatile building blocks, thus providing successful synthesis of all the structures. A blockwise sequence was employed to access targets **2**–**6** whereby each glycosylation product was either deprotected to give the final products or became the acceptor for the next glycosylation step. Linear core 3 trisaccharide **7** was assembled using an orthogonal glycosylation strategy that allowed consecutive glycosylations without the need for intermediate protecting group manipulations. A sequential deprotection strategy that did not require chromatography-based purification between steps was employed, and all unprotected structures were isolated after global deprotection, in yields ranging from 52–70% using C-18 reversed-phase column chromatography purification. All target compounds **1**–**7** were produced functionalized with an amine linker ready to be incorporated onto glycoarray platforms for high throughput biological screening [31].

## Supporting Information

File 1 Experimental Section.
