# Supplementary material for: Synthesis of mucin-type O-glycan probes as aminopropyl glycosides
Source: Beilstein J Org Chem. 2013 Sep 13;9:1867–72. doi: 10.3762/bjoc.9.218 (PMC3778329; doi:10.3762/bjoc.9.218)
Supplement: File 1 — Experimental Section. [file Beilstein_J_Org_Chem-09-1867-s001.pdf]

Supporting Information

for

Synthesis of mucin-type *O*-glycan probes as aminopropyl glycosides

David Benito-Alifonso[1], Rachel A. Jones[1], Anh-Tuan Tran[1], Hannah Woodward[1], Nichola Smith[2] and M. Carmen Galan*[1]

Address: [1]School of Chemistry, University of Bristol, Cantock's Close, Bristol BS8 1TS, UK Fax: (0)1179298611, Tel: (0)1179287654 and [2]Novartis Institute for Biomolecular Research, Novartis Horsham Research Centre, Horsham RH12 5AB, UK

Email: M. Carmen Galan - m.c.galan@bris.ac.uk

* Corresponding author

Experimental Section

General experimental. Chemicals were purchased from Aldrich and Fluka and used without further purification. Preactivated molecular sieves kept in an oven at 150 °C were activated in a standard microwave (800 W) for 3 minutes (3 × 1 minute) and cooled under vacuum. Dry solvents, where necessary, were obtained by distillation using standard procedures or by passage through a column of anhydrous alumina using equipment from Anhydrous

Engineering (University of Bristol) based on the Grubbs' design. Reactions requiring anhydrous conditions were performed under an atmosphere of dry nitrogen; glassware, syringes and needles were either flame dried immediately prior to use or placed in an oven (150 °C) for at least 2 hours and allowed to cool either in a desiccators or under an atmosphere of dry nitrogen; liquid reagents, solutions or solvents were added via syringe or cannula through rubber septa; solid reagents were added via Schlenk type adapters. Typical reactions were carried out on 40−50 mg scale. Reactions were monitored by TLC on Kieselgel 60 F_{254} (Merck). Detection was by examination under UV light (254 nm) and by charring with 10% sulfuric acid in ethanol. Flash chromatography was performed using silica gel [Merck, 230−400 mesh (40−63 μm)], the crude material was applied to the column as a solution in CH_2Cl_2 or by pre-adsorption onto silica, as appropriate. Extracts were concentrated under reduced pressure using both a Büchi rotary evaporator (bath temperatures up to 40 °C) at a pressure of either 15 mmHg (diaphragm pump) or 0.1 mmHg (oil pump), as appropriate, and a high vacuum line at room temperature. The unit of the specific rotations (deg·mL)/(g·dm), are implicit and are not included with the reported value. Concentration *c* is given in g/100 mL. ^{1}H NMR and ^{13}C NMR spectra were measured in the solvent stated using Varian INOVA 400 or 500 instruments, respectively. Chemical shifts are quoted in parts per million from $SiMe_4$ and coupling constants (*J*) are given in Hertz. Multiplicities are abbreviated as: b (broad), s (singlet), d (doublet), t (triplet), q (quartet), m (multiplet) or combinations thereof. ^{1}H and ^{13}C assignments were made with data collected in 1D and 2D NMR experiments (COSY, HSQC and HMBC).

3-Chloropropyl 3-*O*-acetyl-4,6-*O*-benzylidene-2-deoxy-2-(2,2,2-trichloroethoxycarbonyl-amino)-α-D-galactopyranoside (9). To a solution of chloride **8** (782 mg, 1.51 mmol) in pyridine (5 mL), Ac_2O (5 mL) was added slowly over 5 minutes. The reaction mixture was stirred at r.t. for 16 h until complete consumption of the starting material as observed by TLC (*n*-hexanes/EtOAc, 1:1, v/v). The reaction mixture was then diluted with CH_2Cl_2 (5 mL) and H_2O (5 mL) and stirred at r.t. for 30 min. The aqueous layer was extracted with CH_2Cl_2 (2 × 5 mL) and the combined chlorinated layers were

washed with HCl (3 × 5 mL, 1 M), $NaHCO_3$ (sat., aq) (5 mL) and brine (5 mL) dried over Na_2SO_4, filtered and the filtrates co-evaporated with toluene under reduced pressure to yield **9** (784 mg, 92%) as a white foam. $[\alpha]_D^{22.5}$ +137 (*c* = 0.015, $CHCl_3$); IR (neat) ν_{max} cm^{-1}; 3336, 2916, 1736 (s, C=O), 1514, 1235, 1037, 698; ^1H NMR (500 MHz, $CDCl_3$) δ_H 7.55 – 7.53 (m, 2H, Ph), 7.39 – 7.36 (m, 3H, Ph), 5.56 (s, 1H, PhC*H*), 5.17 (dd, 1H, $J_{3,2}$ = 11.3 Hz, $J_{3,4}$ = 3.3 Hz, H-3), 5.14 (d, 1H, $J_{NH,2}$ = 10.2 Hz, NH), 5.05 (d, 1H, $J_{1,2}$ = 3.6 Hz, H-1), 4.84 (d, 1H, *J* = 12.1 Hz, C*H*$HCCl_3$), 4.65 (d, 1H, *J* = 12.1 Hz, CH*H*CCl_3), 4.53 (*app* td, 1H, $J_{2,3}$ = 11.3 Hz, $J_{2,NH}$ = 10.0 Hz, $J_{1,2}$ = 3.6 Hz, H-2), 4.37 (dd, 1H, $J_{3,4}$ = 3.3 Hz, $J_{4,5}$ = 1.2 Hz, H-4), 4.29 (dd, 1H, $J_{6a,6b}$ = 12.6 Hz, $J_{5,6a}$ = 1.7 Hz, H-6a), 4.09 (dd, 1H, $J_{6b,6a}$ = 12.6 Hz, $J_{5,6b}$ = 1.7 Hz, H-6b), 3.97 (ddd, 1H, *J* = 9.9 Hz, *J* = 7.3 Hz, *J* = 5.1 Hz, OC*H*H), 3.78 (*br*. s, 1H, H-5), 3.67 (t, 2H, *J* = 6.2 Hz, CH_2Cl), 3.62 (dt, 1H, *J* = 10.0 Hz, *J* = 5.7 Hz, OCH*H*), 2.16 – 2.03 (m, 2H, C$H_2$$CH_2$Cl), 2.09 (s, 3H, CH_3); ^{13}C NMR (126 MHz, $CDCl_3$) δ_C 171.3 (C=O, *Ac*), 154.1 (C=O, *Troc*), 137.4 (C*q*, *aromatic*), 129.1, 128.2, 126.3 (CH, *aromatics*), 100.9 (Ph*C*H), 98.3 (C-1), 95.5 (*C*Cl_3), 74.5 (CH_2, *Troc*), 73.5 (C-4), 69.2 (C-3 and C-6), 64.6 (O*C*$H_2$$CH_2$), 62.8 (C-5), 49.6 (C-2), 41.5 (*C*H_2Cl), 31.9 (*C*$H_2$$CH_2$Cl), 21.0 ($CH_3$); MALDI-TOF *m/z* calculated for $C_{21}H_{26}Cl_4NO_8^+$ $(MH)^+$ calcd.: 560.0, found 559.9; HRMS: (ESI^+) Found: $[M + Na]^+$ 582.0232, $C_{21}H_{25}Cl_4NaNO_8S$ requires 582.0226 (^{35}Cl).

3-Azidopropyl 3-*O*-acetyl-4,6-*O*-benzylidene-2-deoxy-2-(2,2,2-trichloroethoxycarbonyl-amino)-α-D-galactopyranoside (10). To a solution of chloride **9** (803 mg, 1.43 mmol) in anhydrous MeCN (14 mL) was added *n*-TBAI (53 mg, 0.143 mmol) and NaN_3 (930 mg, 14.5 mmol) at r.t. The reaction mixture was then heated at reflux for a 36 h until complete consumption of the starting material, as shown by TLC (*n*-hexanes/EtOAc, 8:2, v/v). The reaction mixture was then allowed to cool to r.t., filtered through Celite® and concentrated under reduced pressure. The residue was dissolved in EtOAc (15 mL) and washed with $Na_2S_2O_3$ (sat. aq.) (3 × 20 mL). The organic layer was dried over Na_2SO_4, filtered, concentrated under reduced pressure and purified by silica gel column chromatography to yield **10** (716 mg, 88%) as white foam. $[\alpha]_D^{23.5}$ +141 (*c* = 0.018, $CHCl_3$); IR (neat) ν_{max} cm^{-1}; 3334, 2916,

2097 (m, N_3), 1737 (s, C=O), 1516 (m), 1236 (s), 1038 (s); ^{1}H NMR (400 MHz, $CDCl_3$) δ_H 7.54 – 7.52 (m, 2H, Ph), 7.40 – 7.36 (m, 3H, Ph), 5.56 (s, 1H, PhC*H*), 5.24 (d, 1H, $J_{NH,2}$ = 10.0 Hz, NH), 5.17 (dd, 1H, $J_{3,4}$ = 11.3 Hz, $J_{3,2}$ = 3.1 Hz, H-3), 5.03 (d, 1H, $J_{1,2}$ = 3.6 Hz, H-1), 4.85 (d, 1H, *J* = 12.1 Hz, C*H*HCCl$_3$), 4.64 (d, 1H, *J* = 12.1 Hz, CH*H*CCl$_3$), 4.53 (*app* td, 1H, $J_{2,3}$ = 11.3 Hz, $J_{2,NH}$ = 10.0 Hz, $J_{1,2}$ = 3.6 Hz, H-2), 4.36 (d, 1H, $J_{4,5}$ = $J_{3,4}$ = 3.1 Hz, H-4), 4.28 (dd, 1H, $J_{6a,6b}$ = 12.5 Hz, $J_{,5,6a}$ = 1.6 Hz, H-6a), 4.09 (dd, 1H, $J_{6b,6a}$ = 12.5 Hz, $J_{5,6b}$ = 1.7 Hz, H-6b), 3.87 (dt, 1H, *J* = 10.1 Hz, *J* = 6.2 Hz, OC*H*H), 3.74 (*br.* s, 1H, H-5), 3.56 (dt, 1H, *J* = 10.1 Hz, *J* = 5.9 Hz, OCH*H*), 3.44 (t, 2H, *J* = 6.5 Hz, C*H*$_2$N$_3$), 2.08 (s, 3H, CH_3), 1.94 – 1.89 (m, 2H, C*H*$_2$CH$_2$Cl); ^{13}C NMR (100 MHz, $CDCl_3$) δ_C 171.3 (C=O, *Ac*), 154.1 (C=O, *Troc*) 137.4 (C_q, *aromatic*), 129.0, 128.2, 126.3 (CH, *aromatics*), 100.8 (Ph*C*H), 98.3 (C-1), 95.5 (*C*Cl$_3$, *Troc*), 74.5 (CH_2, *Troc*), 73.4 (C-4), 69.2 (C-3 and C-6), 65.3 (O*C*H$_2$), 62.8 (C-5), 49.5 (C-2), 48.5 (*C*H$_2$N$_3$), 28.6 (*C*H$_2$CH$_2$N$_3$), 21.0 (CH_3); HRMS: (ESI$^+$) Found: $[M + Na]^+$ 589.0639, $C_{21}H_{25}Cl_3NaN_4O_8S$ requires 589.0630 (^{35}Cl).

3-Azidopropyl 4,6-*O*-benzylidene-2-deoxy-2-(2,2,2-trichloroethoxycarbonylamino)-α-D-galactopyranoside (11). To a suspension of **10** (716 mg, 1.26 mmol) in MeOH (4.8 mL) was added Et_3N (0.9 mL, 6.3 mmol) and H_2O (0.9 mL). The reaction mixture was stirred at r.t. for 24 h until complete deacetylation as observed by TLC (*n*-hexanes/EtOAc, 4:6, v/v). Co-evaporation of the reaction mixture with PhMe (6 × 15 mL) under reduced pressure afforded **11** (644 mg, 97%) as white flakes that were used in following reactions without any further purification. $[\alpha]_D^{23}$ +105 (*c* = 0.010, $CHCl_3$); IR (neat) ν_{max} cm^{-1} 3334, 2917, 2096 (s, N_3), 1729 (s, C=O), 1519, 1096, 1026, 994, 698; ^{1}H NMR (400 MHz, $CDCl_3$) δ_H 7.55-7.49 (m, 2H, Ph), 7.44-7.33 (m, 3H, Ph), 5.60 (s, 1H, PhC*H*), 5.33 (d, 0.7 H, $J_{NH,2}$ = 9.4 Hz, NH), 5.03 (d, 1H, $J_{1,2}$ = 3.4 Hz, H-1), 4.78 (d, 1H, *J* = 12.1 Hz, C*H*HCCl$_3$, *Troc*) 4.72 (d, 1H, *J* = 12.1 Hz, CH*H*CCl$_3$, *Troc*), 4.30 (dd, 1H, $J_{6a,6b}$ = 12.5 Hz, $J_{5,6a}$ = 1.2 Hz, H-6a), 4.27 (d, 1H, $J_{3,4}$ = 3.7 Hz, H-4), 4.19 (ddd, 1H, $J_{2,3}$ = 10.0 Hz, $J_{2,NH}$ = 9.4 Hz, $J_{2,1}$ = 3.4 Hz, H-2), 4.10 (dd, 1H, $J_{5,6b}$ = 1.6 Hz, H-6b), 3.91 – 3.88 (m, 1H, OC*H*HCH$_2$), 3.84 (dd, 1H, $J_{2,3}$ = 10.0 Hz, $J_{3,4}$ = 3.7 Hz, H-3), 3.72 (*br.*

s, 1H, H-5), 3.57 (dt, 1H, *J* = 10.3 Hz, *J* = 5.9 Hz, OCH*H*CH_2), 3.42 (m, 2H, CH_2N_3), 1.91 (m, 2H, OCH_2C*H₂*); ^{13}C NMR (100 MHz, $CDCl_3$) δ_C 155.0 (C=O, *Troc*), 137.3 (C_q, *aromatic*), 129.3, 128.3, 126.3 (CH, *aromatic*), 101.3 (Ph*C*H), 98.4 (C-1), 95.4 (*C*Cl_3, *Troc*), 75.3 (C-4), 74.7 (CH_2, *Troc*), 69.3 (C-6), 68.7 (C-3), 65.3 (O*C*H_2CH_2), 63.1 (C-5), 52.2 (C-2), 48.6 (CH_2N_3), 28.7 (OCH_2*C*H_2); HRMS: (ESI$^+$) Found: $[M + Na]^+$ 547.0525, $C_{19}H_{23}Cl_3NaN_4O_7$ requires 547.0528 (^{35}Cl).

3-Azidopropyl 3,4,6-tri-*O*-acetyl-2-deoxy-2-(2,2,2-trichloroethoxycarbonylamino)-β-D-glucopyranosyl-(1→3)-4,6-*O*-benzylidene-2-deoxy-2-(2,2,2-trichloroethoxycarbonylamino)-α-D-galactopyranoside (14). To a solution of glycosyl donor **12** (43 mg, 0.074 mmol), acceptor **11** (30 mg, 0.057 mmol) and 4 Å mol sieves in CH_2Cl_2 (300 µL) was added at −40 °C, NIS (33 mg, 0.146 mmol) and TMSOTf (29 µL, 10% solution in CH_2Cl_2). The reaction mixture was stirred for 30 minutes until complete consumption of the acceptor, as observed by TLC (PhMe/EtOAc, 8:2, v/v). The reaction mixture was quenched with Et_3N (50 µL) and purified by silica gel column chromatography (PhMe/EtOAc, 9:1 → 7:3 v/v) to afford disaccharide **14** (50 mg, 90%) as a colourless film. $[\alpha]_D^{21.0}$ + 58.8 (*c* = 0.008, $CHCl_3$); ^{1}H NMR (400 MHz, DMSO) δ_H 7.61 (d, 0.8H, $J_{NH,2}$ = 8.5 Hz, NH’), 7.44-7.35 (m, 5H, Ph), 7.24 (d, 0.8H, $J_{NH,2}$ = 7.5 Hz, NH), 5.51 (s, 1H, PhC*H*), 5.09 (t, 1H, $J_{2,3}$ = $J_{3,4}$ = 10.0 Hz, H-3’), 5.08 (d, 1H, *J* = 12.0 Hz, C*H*$HCCl_3$) 5.03 (d, 1H, *J* = 12.0 Hz, C*H*$HCCl_3$), 4.91 (d, 1H, $J_{1,2}$ = 3.0 Hz, H-1), 4.87 (d, 1H, $J_{1',2'}$ = 8.0 Hz, H-1’), 4.86 (dd, 1H, $J_{4,5}$ = 9.5 Hz, $J_{3,4}$ = 10.0 Hz, H-4’), 4.56 (d, 1H, *J* = 12.0 Hz, CH*H*CCl_3), 4.47 (d, 1H, *J* = 12.0 Hz, CH*H*CCl_3), 4.44 (*br*. d, *J* = 3.0 Hz, H-4), 4.20 (dd, 1H, $J_{5,6a}$ = 2.5 Hz, $J_{6a,6b}$ = 12.0 Hz, H-6a’), 4.13 (dd, 1H, $J_{5,6b}$ = 5.0 Hz, $J_{6b,6a}$ = 12.0 Hz, H-6b’), 4.09-3.99 (m, 3H, H-6a, H-6b, H-3), 3.99-3.86 (m, 2H, H-2, H-5’), 3.74-3.69 (m, 2H, OC*H*H, H-5), 3.56-3.47 (m, 1H, H-2’) 3.46 – 3.38 (m, 3H, OCH*H*, C*H₂*N_3), 2.02 (s, 3H, CH_3), 1.98 (s, 3H, CH_3), 1.86 (s, 3H, CH_3), 1.83-1.76 (m, 2H, OCH_2C*H₂*); ^{13}C NMR (100 MHz, DMSO) δ_C 170.0, 169.6, 169.3 (3 × C=O, *Ac*), 154.4, 154.3 (2 × C=O, *Troc*), 138.4 (C*q*, *aromatic*), 128.8, 128.02, 126.2, (CH, *aromatics*), 101.3 (C-1’), 99.9 (Ph*C*H), 97.1 (C-1), 96.3 (2 × *C*Cl_3), 75.3 (C-3), 74.8 (C-4),

73.5, 73.3 (2 × CH_2, *Troc*), 73.1 (C-3'), 70.9 (C-5'), 68.7 (C-6), 68.3 (C-4'), 64.5 (O<u>C</u>H_2), 62.5 (C-5), 61.8 (C-6'), 55.4 (C-2'), 50.4 (C-2), 40.7 (<u>C</u>H_2N_3), 28.3 (OCH_2<u>C</u>H_2), 20.6, 20.4, 20.3 (3 × CH_3); HRMS: (ESI^+) Found: $[M + Na]^+$ 1008.0546, $C_{34}H_{41}Cl_6N_5NaO_{16}$ requires 1008.0572 (^{35}Cl).

3-Aminopropyl 2-acetamido-2-deoxy-β-D-glucopyranosyl-(1→3)-2-acetamido-2-deoxy-2-α-D-galactopyranoside, hydrochloride (2). To a solution of disaccharide **14** (73 mg, 74 μmol) dissolved in THF (2 mL) was added LiOH (1 mL, 1 M). The reaction mixture was stirred at reflux for 18 h until complete hydrolysis of acetate and trichloroethylcarbamate groups was observed by TLC (iPrOH/H_2O/NH_4OH, 7:3:1). The mixture was neutralised with HCl (0.1 M) and the solvent was co-evaporated with toluene under reduced pressure. The dried residue was then re-dissolved in pyridine (3 mL) and Ac_2O (2 mL) and allowed to stir at r.t. for a further 16 h until complete acetylation was observed by TLC (CH_2Cl_2 /MeOH, 9:1). The reaction mixture was concentrated under reduced pressure and the residue was dissolved in MeOH (2 mL) and sodium methoxide (1 mg, 18 μmol) was added. The reaction mixture was stirred at r.t. for 3 h until complete deacetylation as observed by TLC (iPrOH/H_2O/NH_4OH, 7:3:1). The reaction was then neutralised with HCl (0.1 M) and the solvent evaporated under reduced pressure and then re-dissolved in 5% HCl in EtOH (2 mL) and Pd/C (10 mg, 10 wt %) was added. The reaction mixture was placed under an atmosphere of H_2 for 16 h. The mixture was then filtered through Celite® and the filtrate was concentrated under reduced pressure and the dried residue was then purified by C_{18} column chromatography (H_2O →H_2O/MeOH, 19:1, v/v) to yield **2** (23 mg, 60%) as a white foam. $[\alpha]_D^{21}$ +20 (*c* = 0.248, H_2O) ^1H NMR (500 MHz, D_2O) δ_H 4.73 (d, 1H, $J_{1,2}$ = 3.9 Hz, H-1), 4.50 (d, 1H, $J_{1'',2''}$ = 8.4 Hz, H-1''), 4.13 (dd, 1H, $J_{2,3}$ = 11.2 Hz, $J_{1,2}$ = 3.8 Hz, H-2), 4.10 (app d, 1H, $J_{3,4}$ = 3.5 Hz, H-4), 3.89 – 3.82 (m, 2H, H-3, H-4'), 3.78 (dd, 1H, $J_{6a',6b'}$ = 12.5 Hz, $J_{,5,6a'}$ = 1.8 Hz, H-6a'), 3.71 – 3.61 (m, 4H, H-7a, H-6b', H-6a,b), 3.58 (dd, 1H, $J_{2',3'}$ = 10.5 Hz, $J_{1',2'}$ = 8.4 Hz, H-2'), 3.48 – 3.41 (m, 2H, H-3', H-7b), 3.37 – 3.30 (m, 2H, H-5, H-5'), 3.02 (t, 2H, *J* = 7.6 Hz, H-9a,b), 1.94 (s, 3H, CH_3), 1.91 (s, 3H, CH_3), 1.90 – 1.84 (m, 2H, H-8a,b); ^{13}C NMR (126 MHz, D_2O) δ_C

105.0 (C-1'), 99.8 (C-1), 79.0 (C-3), 78.2 (C-5), 76.1 (C-3'), 73.3 (C-4'), 72.4 (C-5'), 71.4 (C-4), 67.6 (C-7), 63.8 (C-6), 63.4 (C-6'), 58.2 (C-2'), 51.2 (C-2), 39.9 (C-9), 29.5 (C-8), 25.0 (CH_3), 24.8 (CH_3). HRMS: (ESI^+) Found: $[M + H]^+$ 504.2164, $C_{19}H_{35}N_3O_{11}Na$ requires 504.2169.

3-Azidopropyl 2,3,4,6-tetra-*O*-acetyl-β-D-galactopyranosyl-(1→3)-4,6-*O*-benzylidene-2-deoxy-2-(2,2,2-trichloroethoxycarbonylamino)-α-D-galactopyranoside (15). To a solution of peracteylated glycosyl donor **13** (71 mg, 0.145 mmol), acceptor 11 (54 mg, 0.10 mmol) and 4 Å mol sieves in CH_2Cl_2 (700 µL) was added at −40°C TMSOTf (25 µL, 10% solution in CH_2Cl_2, 0.13 equiv). The mixture was then stirred for 2 h. The reaction was quenched with Et_3N (50 µL) and purified by silica gel column chromatography (petrol ether/EtOAc, 55:45, v/v) to afford disaccharide **15** (72 mg, 76%). ^{1}H NMR (400 MHz, $CDCl_3$) δ_H 7.56 – 7.54 (m, 2H, Ph), 7.39 – 7.34 (m, 3H, Ph), 5.56 (s, 1H, PhC$\underline{H}$), 5.40 (*br.* d, 1H, $J_{4,3}$ = 3.5, H-4'), 5.26 (d, 1H, $J_{NH,2}$ = 9.0 Hz, NH), 5.24 (dd, 1H, $J_{2,1}$ = 8.0 Hz, $J_{2,3}$ = 10.5 Hz, H-2'), 5.07 (d, 1H, $J_{1,2}$ = 3.5, Hz, H-1), 4.99 (dd, 1H, $J_{3,4}$ = 3.5 Hz, $J_{3,2}$ = 10.5 Hz, H-3'), 4.85 (d, J = 12.0 Hz, C$\underline{H}$HCCl$_3$), 4.76 (d, 1H, $J_{1,2}$ = 8.0 Hz, H-1'), 4.63 (d, 1H, J = 12.0 Hz, CH$\underline{H}$CCl$_3$), 4.41 (ddd, 1H, $J_{1,2}$ = 3.5 Hz, $J_{2,NH}$ = 9.0 Hz, $J_{2,3}$ = 11.0 Hz, H-2), 4.35 (*br.* d, $J_{3,4}$ = 3.0 Hz, H-4), 4.29 (*br.* d, 1H, $J_{6a,6b}$ = 12.0 Hz, H-6a), 4.21 (dd, 1H, $J_{5,6a}$ = 6.5 Hz, $J_{6a,6b}$ = 11.5 Hz, H-6a'), 4.13 (dd, 1H, $J_{5,6b}$ = 6.5 Hz, $J_{6b,6a}$ = 11.5 Hz, H-6b'), 4.07 (*br.* d, $J_{6b,6a}$ = 12.0 Hz, H-6b), 4.01 (dd, 1H, $J_{3,4}$ = 3.0, $J_{2,3}$ = 10.5 Hz, H-3), 3.94 (t, 1H, $J_{5,6a}$ = $J_{5,6b}$ = 6.5 Hz, H-5'), 3.89 – 3.83 (m,1H, OC$\underline{H}$H), 3.68 (*br* s, 1H, H-5), 3.57 (dt, 1H, J = 10.5 Hz, J = 6.5 Hz, OCH$\underline{H}$), 3.42 (t, 2H, J = 6.5 Hz, C$\underline{H_2}$N$_3$), 2.16 (s, 3H, CH_3), 2.06 (s, 3H, CH_3), 2.05 (s, 3H, CH_3), 1.98, (s, 3H, CH_3), 1.98 – 1.87 (m, 2H, OCH$_2$C$\underline{H_2}$). ^{13}C NMR (100 MHz, $CDCl_3$) δ_C 170.3, 170.3,170.1, 169.4 (4 × C=O, *Ac*), 153.9 (CO, *Troc*), 137.5 (C*q*, *aromatic*), 129.0 – 126.2 (CH, *aromatics*), 101.3 (C-1'), 100.7 (Ph$\underline{C}$H), 98.2 (C-1), 95.4 (CCl_3, *Troc*), 75.2 (C-4), 74.5 (CH_2, *Troc*), 74.2 (C-3), 70.9 (C-5'), 70.8 (C-3'), 69.2 (C-6), 68.6 (C-2'), 66.8 (C-4'), 65.5 (O$\underline{C}$H$_2$), 63.2 (C-5), 61.3 (C-6'), 50.5 (C-2), 49.7 ($\underline{C}$H$_2$N$_3$), 28.4 (OCH$_2\underline{C}$H$_2$), 20.8, 20.7, 20.6, 20.5 (4 × CH_3); HRMS: (ESI^+) Found: $[M + Na]^+$ 877.1448, $C_{34}H_{41}Cl_3N_4NaO_{16}$ requires 877.1448 (^{35}Cl).

3-Aminopropyl β-D-galactopyranosyl-(1→3)-2-acetamido-2-deoxy-2-α-D-galactopyranoside, hydrochloride (3). To a solution of disaccharide **15** (45 mg, 53 µmol) in THF (4 mL) was added LiOH (800 µL, 1 M). The reaction mixture was stirred at reflux for 18 h when it was allowed to cool to r.t. The mixture was then neutralised with HCl (0.1 M) and the solvent was co-evaporated with toluene under reduced pressure. The dried residue was re-dissolved in pyridine (2 mL) and Ac_2O (1 mL) and was allowed to stir at r.t. for a further 16 h until complete acetylation was observed by TLC (CH_2Cl_2 /MeOH, 9:1). The reaction mixture was then concentrated under reduced pressure and without further purification, the crude mixture was then dissolved in 5% HCl in EtOH (5 mL) and Pd/C (4 mg, 10 wt %) was added to the reaction mixture. It was then placed under an atmosphere of H_2 and stirred at r.t. for 48 h. The reaction mixture was then filtered through Celite® which was washed thoroughly with EtOH. The combined washings were concentrated under reduced pressure and purified by C_{18} column chromatography (H_2O → H_2O/MeOH, 8:2, v/v) to yield the hydrochloride salt of **3** (13 mg, 70%) as a white solid. $[\alpha]_D^{21}$ + 20 (*c* = 0.006, H_2O); ^1H NMR (500 MHz, D_2O) 4.87 (d, 1H, $J_{1,2}$ = 3.5, Hz, H-1), 4.44 (d, 1H, $J_{1,2}$ = 8.0 Hz, H-1'), 4.30 (dd, 1H, $J_{1,2}$ = 3.5 Hz, $J_{2,3}$ = 11.0 Hz, H-2), 4.20 (*br*. d, 1H, $J_{4,3}$ = 3.0 Hz, H-4), 4.00 (dd, 1H, $J_{3,4}$ = 3.0, $J_{2,3}$ = 10.5 Hz, H-3), 3.94(dd, 1H, $J_{5,6a}$ = 4.5 Hz, $J_{5,6b}$ = 7.5 Hz, H-5), 3.88 (*br*. d, 1H, $J_{3,4}$ = 3.0 Hz, H-4'), 3.78 – 3.76 (m, 1H, OC<u>*H*</u>H), 3.74 – 3.67 (m, 4H, H-6a, H-6b, H-6a', H-6b'), 3.63 (dd, 1H, $J_{5,6a}$ = 4.5 Hz, $J_{5,6b}$ = 8.0 Hz, H-5'), 3.59 (dd, 1H, $J_{3,4}$ = 3.0 Hz, $J_{2,3}$ = 10.0 Hz, H-3'), 3.56-3.52 (m, 1H, OCH<u>*H*</u>), 3.10 (t, 2H, *J* = 7.5 Hz, $C\underline{H_2}NH_2$), 1.99 (s, 3H, CH_3), 1.99 – 1.95 (m, 2H, $OCH_2C\underline{H_2}$), ^{13}C NMR (100 MHz, D_2O) 174.1 (C=O), 105.3 (C-1'), 97.9 (C-1), 77.7 (C-3), 75.6 (C-5'), 73.1 (C-3'), 71.4 (C-2'), 71.2 (C-5), 69.4 (C-4), 69.2 (C-4'), 65.6 ($O\underline{C}H_2$), 61.9, 61.7 (C-6 and C-6'), 49.2 (C-2) 37.8 ($\underline{C}H_2NH_2$), 27.4 ($OCH_2\underline{C}H_2$), 22.6 ($O\underline{C}H_3$); HRMS: (ESI^+) Found: $[M + H]^+$ 441.2086, $C_{17}H_{33}N_2NaO_{11}$ requires 441.2079.

3-Azidopropyl 2,3,4,6-tetra-*O*-acetyl-β-D-glucopyranosyl-(1→3)-3,4,6-tri-*O*-acetyl-2-deoxy-2-(2,2,2-trichloroethoxycarbonylamino)-β-D-glucopyranosyl-(1→6)-2-deoxy-2-(2,2,2-trichloroethoxycarbonylamino)-α-D-galactopyranoside (17). To a solution of disaccharide **16** (75 mg, 0.086 mmol), glycosyl donor 12 (80 mg, 0.128 mmol) in anhydrous CH_2Cl_2 (1.5 mL) was added activated 4 Å MS (150 mg). The mixture was stirred at r.t. for 1 h under an atmosphere of nitrogen. The mixture was cooled to −78 °C and TMSOTf (3.4 μL, 0.017 mmol) was added with stirring. The reaction was kept at −78 °C for 3 h and then quenched with Et_3N (50 μL). The reaction was filtered through celite and washed with CH_2Cl_2. The combined filtrate and CH_2Cl_2 washings were washed with water, dried over anhydrous Na_2SO_4, and concentrated under reduced pressure. The residue was purified by silica gel column chromatography (hexane/EtOAc, 1:1 to 1:2) to afford **17** (69 mg, 66%). $[\alpha]_D^{21}$ +27 (*c* = 0.002, CH_2Cl_2); ^{1}H NMR (400 MHz, $CDCl_3$) δ = 5.37 (dd, $J_{4',3'}$ = 3.6 Hz, 1.2, 1H, H-4'), 5.27 (app t, $J_{3'',4''}$ = $J_{2'',3''}$ = 9.5 Hz, 1H, H-3''), 5.20 (dd, $J_{2',3'}$ = 10.5 Hz, $J_{1',2'}$ = 7.9, 1H, H-2'), 5.06 (app t, $J_{3',4''}$ = $J_{4'',5''}$ = 9.6, 1H, H-4''), 4.97 (dd, $J_{2',3'}$ = 10.5 Hz, $J_{3',4'}$ = 3.4 Hz, 1H, H-3'), 4.90 (d, *J* = 12.1 Hz, 1H, C*H*HCl_3), 4.86 – 4.77 (m, 2H, H-1, CH*H*Cl_3), 4.70 – 4.66 (m, 2H, H-1'', C*H*HCl_3), 4.64 – 4.55 (m, 2H, H-1', CH*H*Cl_3), 4.33 – 4.00 (m, 7H, H-2, 2 × H-6', 2 × H-6'', H-6a, H-5), 3.97 – 3.87 (m, 2H, H-5', H-5''), 3.84 – 3.67 (m, 3H,OC$\underline{H}$H, H-3, H-4, H-2''), 3.55 – 3.47 (m, 1H, OCH$\underline{H}$), 3.43 (t, *J*=6.4, 2H, CH_2N_3), 2.16 (s, 3H), 2.09 (s, 3H), 2.06 (s, 3H), 2.04 (s, 3H), 2.02 (s, 3H), 2.01 (s, 3H), 1.97 (s, 3H), 1.94 – 1.86 (m, 2H, C$\underline{H_2}$$CH_2N_3$); ^{13}C NMR (101 MHz, $CDCl_3$) δ 170.4 (C=O), 169.6 (C=O), 101.6 (C-1'), 101.1 (C-1''), 97.6 (C-1), 95.4 (CCl_3), 78.2 (C-3), 74.6 (CH_2, *Troc*), 74.3 (CH_2, *Troc*), 71.9 (C-3''), 71.8 (C-4), 70.9 (C-5'), 70.5 (C-3'), 69.1 (C-5''), 68.5 (C-4''), 68.3 (C-2'), 67.9 (C-5), 66.8 (C-4'), 65.5 (C-2''), 65.5 (OCH_2), 61.9 (C-6), 61.4 (C-6), 61.4 (C-6), 50.2 (C-2), 48.8 (CH_2N_3), 28.5 ($\underline{C}H_2CH_2N_3$), 20.7 (CH_3), 20.6 (CH_3), 20.6 (CH_3), 20.6 (CH_3), 20.5 (CH_3). HRMS: (ESI^+) Found: $[M + H]^+$ 1227.1320, $C_{41}H_{55}Cl_6N_5O_{25}$ requires 1227.1317 (^{35}Cl).

3-Aminopropyl β-D-glucopyranosyl-(1→3)-2-acetamido-2-deoxy-2-β-D-glucopyranosyl-(1→6)-2-acetamido-2-deoxy-2-α-D-galactopyranoside, hydrochloride (4). To a solution of trisaccharide **17** (22 mg, 0.018 mmol) in THF (0.5 mL) was added LiOH (0.2 mL, 1 M). The reaction mixture was stirred at reflux for 18 h until complete hydrolysis of acetates and trichlorocarbamate groups was observed by TLC (iPrOH/H_2O/NH_4OH, 7:3:1). The mixture was then neutralised with HCl (0.1 M) and the solvent was co-evaporated with toluene under reduced pressure. The dried residue was re-dissolved in pyridine (2 mL) and Ac_2O (1 mL) and was allowed to stir at r.t. for a further 16 h until complete acetylation was observed by TLC (CH_2Cl_2/MeOH, 9:1). The reaction mixture was concentrated under reduced pressure and the dried residue was re-dissolved in MeOH (2 mL) and sodium methoxide (0.1 mg, 0.002 mmol) was added and the reaction mixture was stirred at r.t. for 3 h until complete deacetylation was observed by TLC (iPrOH/H_2O/NH_4OH, 7:3:1). The reaction was neutralised with HCl (0.1 M) and the solvent evaporated under reduced pressure and then redissolved in 5% HCl in EtOH (2 mL). Pd/C (10 mg, 10 wt %) was added to the reaction mixture and it was placed under an atmosphere of H_2 for 16 h. The reaction mixture was then filtered through Celite® and the filtrate was concentrated under reduced pressure and purified by C_{18} column chromatography (H_2O → H_2O/MeOH, 19:1, v/v) to yield the hydrochloride salt of trisaccharide **4** (7.4 mg, 64%) as a white foam. $[\alpha]_D^{21}$ +38 (*c* = 0.003, H_2O) ^{1}H NMR (500 MHz, D_2O) δ_H 4.76 (d, 1H, $J_{1,2}$ = 3.8 Hz, H-1), 4.40 (d, 1H, $J_{1'',2''}$ =8.5 Hz, H-1''), 4.33 (d, 1H, $J_{1',2'}$ = 7.8 Hz, H-1'), 4.20 (dd, 1H, $J_{2,3}$ = 11.1 Hz, $J_{1,2}$ = 3.7 Hz, H-2), 4.11 (app d, 1H, $J_{3',4'}$ = 2.6 Hz, H-4'), 3.97 – 3.76 (m, 5H, H-2'', H-3'', H-3, H-5', H-6*, H-4), 3.72 – 3.55 (m, 5H, OCH_2, H-2', 3 × H-6*), 3.54 – 3.47 (m, 2H, H-5, H-3'), 3.44 – 3.28 (m, 8H, OCH_2, H-5'', H-4'', 2xH-6*, CH_2NH_2), 1.89 (s, 3H, CH_3), 1.89 (s, 3H, CH_3), 1.82 – 1.73 (m, 2H, $OCH_2C\underline{H}_2$). (H-6* = H-6a, H-6b, H-6a', H-6b', H-6a'' or H6b'') ^{13}C NMR (126 MHz, D_2O) δ 174.4 (C=O), 174.4 (C=O),97.0 (C-1), 101.4 (C-1''), 104.4 (C-1'), 48.6 (C-2), 68.7 (C-4'), 77.0 (C-2''), 76.9 (C-3''), 70.5 (C-3), 68.8 (C-5''), 60.6 (C-6), 68.4 (C-4), 64.8 (OCH2), 60.9 (C-6), 64.6 (C-6), 55.4 (C-2'), 74.9 (C-5), 72.4 (C-3'), 73.8 (C-5''), 70.5 (C-4''), 48.1 (CH_2NH_2), 28.1 (CH_3), 26.5 (CH_3), 28.4

($CH_2CH_2NH_2$). HRMS: (ESI^+) Found: $[M + H]^+$ 644.2884, $C_{41}H_{56}Cl_6N_5O_{25}$ requires 644.2873 (^{35}Cl).

3-Azidopropyl 2-Acetamido-2-deoxy-3,4-O-isopropylidene-D-galactopyranoside (19). To a solution of **18** (495 mg, 1.63 mmol) in a mixture of DMF (4.7 mL) and 2,2 dimethoxypropane (9.4 mL) was added *p*-TsOH monohydrate (31 mg, 0.16 mmol) at r.t. The solution was stirred at 65 °C for 5 h after which the mixture was cooled to r.t., and Et_3N (500 μL) was added. The mixture was stirred for another 15 min and the solvent was co-evaporated with toluene (2 × 5 mL) under reduced pressure. The dried residue was dissolved in MeOH/H_2O (10:1) (15 mL) and heated under reflux for 30 min until TLC (CH_2Cl_2/MeOH 9:1) showed the complete disappearance of the reaction intermediate. The solution was then co-evaporated with toluene under reduced pressure and the dried residue was purified by silica gel column chromatography (PhMe/acetone 6:4 to 5:5) to give **19** (432 mg, 77%) as off-white foam. $[\alpha]_D^{21}$ +112 (*c* = 0.006, CH_2Cl_2); ^1H NMR (400 MHz, $CDCl_3$) δ_H 5.89 (d, 1H, $J_{NH,2}$ = 9.1 Hz, NH), 4.76 (d, 1H, $J_{1,2}$ = 3.3 Hz, H-1), 4.20 (td, 1H, $J_{2,3}$ = 9.2 Hz, $J_{1,2}$ = 3.2 Hz, H-2), 4.15 (dd, 1H, $J_{4,3}$ = 5.0 Hz, $J_{4,5}$ = 2.4 Hz, H-4), 4.08 – 4.03 (dd, 1H, $J_{2,3}$ = 9.2 Hz, $J_{3,4}$ = 5.0 Hz, H-3), 3.98 (ddd, 1H, $J_{5,6a}$ = 6.7 Hz, $J_{5,6b}$ = 4.4 Hz, $J_{4,5}$ = 2.4 Hz, H-5), 3.94 – 3.87 (m, 1H, H-6a), 3.83 – 3.72 (m, 2H, H-6b, OC$\underline{H}$HCH_2), 3.44 (dt, 1H, *J*=10.1, 6.1, OCH$\underline{H}$$CH_2$), 3.39 – 3.30 (m, 2H, $CH_2C\underline{H}_2N_3$), 1.98 (s, 3H, CH_3), 1.84 (dt, 2H, *J*=12.0, 6.1, $OCH_2C\underline{H}_2$), 1.51 (s, 3H, CH_3), 1.29 (s, 3H, CH_3). ^{13}C NMR (101 MHz, $CDCl_3$) δ_C 170.4 (C=O), 109.9 ($\underline{C}CH_3CH_3$), 97.9 (C-1), 74.4 (C-3), 73.4 (C-4), 68.0 (C-5), 65.2 (O$\underline{C}H_2$), 62.6 (C-6), 50.6 (C-2), 48.6 ($\underline{C}H_2N_3$), 28.5 ($OCH_2\underline{C}H_2$), 28.0 (CH_3), 26.6 (CH_3), 23.4 (CH_3) HRMS: (ESI^+) Found: $[M + Na]^+$ 367.1588, $C_{25}H_{46}N_3O_{16}Na$ requires 367.1594.

3-Azidopropyl [3',4',6'-tri-O-acetyl-2'-deoxy-2'-acetamido-D-glucopyranosyl]-(1→6)-α-D-3,4-di-*O*-acetyl-2-deoxy-2-acetamido-D-galactopyranoside (20b). To a solution of isopropylidene galactosamine derivative **19** (54 mg, 0.16 mmol) in a mixture of anhydrous CH_2Cl_2/MeCN (2:1, 3 mL) under N_2 was added glycosyl donor **12** (147 mg, 0.24 mmol) and 4

Å MS (80 mg). The reaction mixture was stirred at r.t. for 1 h before being cooled to −40 °C. TMSOTf (3 µL, 0.016 µmol) was added and the reaction mixture was stirred for 4 h at −40 °C until complete consumption of the starting material, as observed by TLC (PhMe/acetone/MeOH, 7:2:1, v/v/v). The reaction mixture was quenched with Et_3N (50 µL), concentrated and purified by silica gel column chromatography (CH_2Cl_2 → CH_2Cl_2/MeOH, 95:5, v/v) to yield an inseparable mixture of disaccharide **20a** along with galactosamine acceptor **18**. The mixture was then dissolved in THF (4 mL) and NaOH (1 M, 1mL) and stirred at 60 ºC for 4 h. After neutralising with HCl (0.1 M) the reaction mixture was co-evaporated with PhMe (3 × 3 mL) under reduced pressure and the dried residue was re-dissolved in pyridine (2 mL) and Ac_2O (1 mL) and allowed to stir at r.t. for a further 16 h until complete acetylation was observed by TLC (CH_2Cl_2/MeOH, 9:1). The reaction mixture was then diluted with CH_2Cl_2 and washed with HCl (0.1 M, 2 × 50 mL), aqueous $NaHCO_3$ (sat.), dried over anhydrous Na_2SO_4, filtered and the solvent evaporated under reduced pressure. The dried residue was purified by silica gel column chromatography (CH_2Cl_2 → CH_2Cl_2/MeOH, 95:5, v/v) to afford disaccharide **20b** (46 mg, 41% over 3 steps). $[\alpha]_D^{21}$ +43 (*c* = 0.006, CH_2Cl_2) ^{1}H NMR (500 MHz, $CDCl_3$) δ_H 5.69 – 5.65 (m, 2H, NH), 5.34 – 5.27 (m, 2H, H-4, H-4'), 5.11 (dd, 1H, *J*=11.3, 3.3, H-3), 5.03 (t, 1H, *J*=9.6, H-3'), 4.83 (d, 1H, $J_{1,2}$ = 3.6 Hz, H-1), 4.71 (d, 1H, $J_{1',2'}$ = 8.2 Hz, H-1'), 4.54 (ddd, 1H, $J_{2,3}$ = 11.3 Hz, $J_{2,NH}$ = 9.6 Hz, $J_{1,2}$ = 3.6 Hz, H-2), 4.26 (dd, 1H, $J_{6a',6b'}$ = 12.3 Hz, $J_{5,6a'}$ = 4.7 Hz, H-6a'), 4.11 – 4.07 (m, 2H, H-6b', H-5'), 3.84 – 3.77 (m, 2H, H-6a, OC*H*H), 3.75 – 3.66 (m, 2H, H-2', H-5), 3.55 (dd, 1H, $J_{6a,6b}$ = 10.4 Hz, $J_{5,6a}$ = 7.4 Hz, H-6b), 3.51 – 3.41 (m, 3H, OCH*H*, CH_2N_3), 2.13 (s, 3H, CH_3), 2.07 (s, 3H, CH_3), 2.00 (s, 3H, CH_3), 1.97 (s, 3H, CH_3), 1.95 (s, 3H), 1.94 – 1.87 (m, 5H, CH_3, $OCH_2C\underline{H_2}$). ^{13}C NMR (126 MHz, $CDCl_3$) δ_C 171.1 (C=O), 170.9 (C=O), 170.8 (C=O), 170.4 (C=O), 170.4 (C=O), 170.2 (C=O), 169.5 (C=O), 100.6 (C-1'), 97.8 (C-1), 72.1 (C-4'), 71.9 (C-5), 68.7 (C-3), 68.6 (C-3'), 68.0 (C-5'), 67.9 (C-4), 67.8 (C-6), 65.4 (OCH_2), 62.1 (C-6'), 55.0 (C-2'), 48.7 (CH_2N3), 48.0 (C-2), 28.7 ($OCH_2\underline{C}H_2$), 23.4 (CH_3), 20.9 (CH_3), 20.9 (CH_3), 20.9 (CH_3), 20.8 (CH_3), 20.7 (CH_3). Found: $[M + Na]^+$ 740.2597, $C_{29}H_{43}N_5O_{16}Na$ requires 740.2603

3-Aminopropyl 2'-deoxy-2'-acetamido-D-glucopyranosyl-(1→6)-α-D-2-deoxy-2-acetamido-D-galactopyranoside hydrochloride (5). To a solution of disaccharide **20b** (46 mg, 0.064 mmol) in THF (1 mL) was added LiOH (0.4 mL, 1 M). The reaction mixture was stirred at reflux for 18 h until complete hydrolysis of acetates and trichlorocarbamate groups was observed by TLC (iPrOH/H_2O/NH_4OH, 7:3:1). The mixture was then neutralised with HCl (0.1 M) and the solvent was co-evaporated with toluene under reduced pressure. The dried residue was re-dissolved in pyridine (2 mL) and Ac_2O (1 mL) and was allowed to stir at r.t. for a further 16 h until complete acetylation was observed by TLC (CH_2Cl_2 /MeOH, 9:1). The reaction mixture was concentrated under reduced pressure and the dried residue was re-dissolved in MeOH (2 mL) was added sodium methoxide (0.3 mg, 6 μmol) and the reaction mixture was stirred at r.t. for 3 h until complete deacetylation as observed by TLC (iPrOH/H_2O/NH_4OH, 7:3:1). The reaction was neutralised with HCl (0.1 M) and the solvent evaporated under reduced pressure and the dried residue was re-dissolved in 5% HCl in EtOH (2 mL). Pd/C (20 mg, 10 wt %) was added to the reaction mixture and it was placed under an atmosphere of H_2 for 16 h. The reaction mixture was then filtered through Celite®. The filtrate was concentrated under reduced pressure and purified by C_{18} column chromatography (H_2O → H_2O/MeOH, 19:1, v/v) to yield the hydrochloric salt of disaccharide **5** (20 mg, 63%) as white foam. $[\alpha]_D^{21}$ −50(c = 0.0002, H_2O); ^{1}H NMR (500 MHz, D_2O) δ_H 4.75 (d, 1H, $J_{1,2}$ = 3.7 Hz, H-1), 4.39 (d, 1H, $J_{1',2'}$ = 8.5 Hz, H-1'), 4.02 (dd, $J_{2,3}$ = 11.0 Hz, $J_{2,1}$ = 3.8 Hz, 1H, H-2), 3.96 (dd, 1H, $J_{6a,6b}$ = 10.8 Hz, $J_{5,6a}$ = 3.0 Hz, H-6a), 3.90 – 3.75 (m, 4H, H-5, H-4, H-6'a, H-3), 3.65 – 3.55 (m, 4H, H-6'b, OC<u>*H*</u>H, H-2', H-6b), 3.45 – 3.28 (m, 4H, H-4', OCH<u>*H*</u>, H-5', H-3'), 2.99 (t, 2H, J=7.6 Hz, C<u>*H*$_2$</u>NH$_2$), 1.91 (s, 3H, CH_3), 1.89 (s, 3H, CH_3), 1.88 – 1.81 (m, 2H, OCH$_2$C<u>*H*$_2$</u>). ^{13}C NMR (126 MHz, D_2O) δ_C 174.5 (C=O), 174.3 (C=O), 101.5 (C-1'), 96.9 (C-1), 75.8 (C-5'), 73.8 (C-4'), 70.1 (C-6), 69.9 (C-3'), 69.6 (C-5), 68.6 (C-4), 67.4 (C-3), 64.6 (OCH_2), 60.6 (C-6'), 54.3 (C-2'), 49.7 (C-2), 37.2 (CH_2NH_2), 26.6 (OCH$_2$<u>C</u>H$_2$), 22.2 (CH_3), 21.8 (CH_3). Found: $[M + H]^+$ 482.2344, $C_{19}H_{36}N_3O_{11}$ requires 482.2344.

3-Azidopropyl [methyl 4,7,8,9-tetra-O-acetyl-3,5-dideoxy-5-acetamido-D-glycero-D-galacto-non-2-ulopyranosyl]onate-(2→6)-*O*-2-acetamido-2-deoxy-D-galactopyranoside (22b). To a solution of adamantyl thioglycoside **21** (280 mg, 0.44 mmol) and isopropylidene galactoside **19** (100 mg, 0.29 mmol) in anhydrous CH_3CN (1.5 mL) was added activated 4 Å MS (100 mg) and the mixture was left stirring at r.t. for 12 h under an atmosphere of nitrogen. The mixture was cooled to −35 °C and NIS (131 mg, 0.58 mmol) and TMSOTf (16 μL, 0.09 mmol) were added with stirring. The reaction was kept at −35 °C for 2 h and then quenched with the addition of Et_3N (50 μL). The reaction was filtered through Celite® and washed with CH_2Cl_2. The combined filtrate and washings were washed with aqueous $Na_2S_2O_3$ and water, dried over anhydrous Na_2SO_4, and concentrated under vacuum. The residue was filtered through a silica column (CH_2Cl_2 /MeOH 19:1) to afford **22a**, which was, without further purification, re-dissolved in MeOH (2 mL) and *p*-TsOH monohydrate (5.5 mg, 0.03 mmol) was added. The reaction mixture was sonicated at r.t. for 15 min and then Et_3N was added to neutralise the acid and the mixture was concentrated under vacuum to yield compound **22b** as an α/β (3/1) mixture. The mixture was purified by silica gel column chromatography (PhMe/acetone/MeOH, 7:2:1) to afford **22b** (90 mg (α-anomer), 40% over two steps) as white foam. ^{1}H NMR (400 MHz, CD_3OD) δ = 5.43 (dd, 1H, $J_{7',8'}$ = 4.8 Hz, $J_{6',7'}$ = 2.2 Hz, H-7'), 5.33 (ddd, 1H, $J_{8', 9b'}$ =7.2, $J_{7',8'}$ = 4.8 Hz, $J_{8', 9a'}$ =2.5, H-8'), 5.22 (td, 1H, $J_{3',4'}$ = 11.0 Hz, $J_{3'eq, 4'}$ = 4.9, H-4'), 4.81 (d, 1H, $J_{1,2}$ = 3.7 Hz, H-1), 4.75 (dd, 1H, $J_{9a', 9b'}$ = 12.4 Hz, $J_{8',9a'}$ = 2.5 Hz, H-9'a), 4.31 – 4.25 (m, 2H, H-2, H-6'), 4.13 (dd, 1H, $J_{9b', 9a'}$ = 12.4 Hz, $J_{8',9b}$ = 7.0 Hz, H-9'b), 4.02 – 3.90 (m, 2H, H-4, H-5'), 3.85 – 3.70 (m, 6H, H-3, OCH_3, H-5, OC*H*HCH_2, H-6a), 3.53 – 3.43 (m, 4H, OCH*H*CH_2, H-6b, $CH_2CH_2N_3$), 2.46 (dd, 1H, $J_{3'eq, 3'ax}$ = 12.9 Hz, $J_{3'eq, 4'}$ = 4.9, H-3'$_{eq}$), 2.11 (s, 3H, CH_3), 2.07 (s, 3H, CH_3), 2.01 (s, 3H, CH_3), 2.00 (s, 3H, CH_3), 1.98 (s, 3H, CH_3), 1.93 – 1.78 (m, 6H, CH_3, H-3'$_{ax}$, OCH_2CH_2). ^{13}C NMR (101 MHz, CD_3OD) δ = 173.9 (C=O), 173.5 (C=O), 172.5 (C=O), 172.4 (C=O), 171.9 (C=O), 171.7 (C=O), 168.8 (C=O), 99.8 (C-2'), 99.1 (C-1), 72.9 (C-8'), 72.3 (C-6'), 70.4 (C-4'), 70.0 (C-5), 69.9 (C-4), 69.7 (C-7'), 69.5 (C-3), 66.1 (OCH_2CH_2), 63.6 (C-6), 63.6 (C-9'), 53.3 (OCH_3), 51.5 (C-2), 50.2 (C-5'), 49.5 ($CH_2CH_2N_3$), 38.4 (C-3'),

29.9 (OCH_2CH_2), 22.7 (CH_3), 22.7 (CH_3), 21.1 (CH_3), 20.8 (CH_3), 20.7 (CH_3), 20.9 (CH_3). HRMS: (ESI^+) Found: $[M + H]^+$ 777.2914, $C_{31}H_{47}N_5O_{18}$ requires 777.2916. $C_{31}H_{47}N_5O_{18}$

3-Aminopropyl-[(3,5-dideoxy-5-acetamido)-D-glycero-D-galacto-non-2-ulosonic acid]-(2→6)-O-2-acetamido-2-deoxy-D-galactopyranoside, hydrochloride (6). To a solution of disaccharide **22b** (20 mg, 0.026 mmol) dissolved in MeOH (2 mL) was added sodium methoxide (0.2 mg, 0.003 mmol) and the reaction mixture stirred at r.t. for 2 h until complete deacetylation was observed by TLC (iPrOH/H_2O/NH_4OH, 7:3:1). The reaction was neutralised with HCl (0.1 M) and the solvent evaporated under reduced pressure. The residue was dissolved THF (1 mL) and LiOH (1 mL, 1 M) was added. The reaction mixture was stirred at r.t. for 5 h until complete hydrolysis of the methyl ester was observed by TLC (iPrOH/H_2O/NH_4OH, 7:3:1). The reaction was neutralised with HCl (0.1 M) and concentrated under reduced pressure. The dried residue was immediately re-dissolved in H_2O (1 mL), Pd/C (6 mg, 10 wt %) was added to the reaction mixture and the reaction was placed under an atmosphere of H_2 for 16 h. The reaction mixture was then filtered through Celite® and freeze-dried. The residue obtained was purified by C_{18} column chromatography (H_2O → H_2O/MeOH, 19:1, v/v) to yield the hydrochloric salt of disaccharide **6** (14.5 mg, 80%) as white foam. $[\alpha]_D^{21}$ +26 (*c* = 0.002, H_2O); ^{1}H NMR (500 MHz, D_2O) δ = 4.77 (d, 1H, $J_{1,2}$ = 3.9 Hz, H-1), 4.04 (dd, 1H, $J_{2,3}$ = 11.1 Hz, $J_{1,2}$ = 3.8, H-2), 3.99 – 3.90 (m, 3H, H-4', H-7', H-8'), 3.78 (dd, 1H, $J_{2,3}$ = 11.1 Hz, $J_{3,4}$ = 3.2, H-3), 3.75 – 3.70 (m, 4H, H-4, H-6a, H-5', H-6'), 3.68 – 3.59 (m, 2H, H-9a', OC*H*HCH$_2$), 3.53 (dd, *J*=12.3, 6.0, 1H, H-6b), 3.48 – 3.38 (m, 2H, OC*H*HCH$_2$, H-5), 3.28 (dd, 1H, $J_{9b', 9a'}$ = 9.8 Hz, $J_{8',9b'}$ = 5.6 Hz, H-9b'), 2.90 (t, 2H, *J*=7.4, C$\underline{H_2}$NH$_2$), 2.25 (dd, 1H, $J_{3eq',3ax'}$ = 13.1 Hz, $J_{3eq',4'}$ = 5.0, H-3eq'), 1.92 (s, 3H, CH_3), 1.91 (s, 3H, CH_3), 1.86 – 1.77 (m, 2H, OCH_2CH_2), 1.51 (dd, 1H, $J_{3ax',3eq',}$ = 13.1 Hz, $J_{3ax',4'}$ = 11.4, H-3ax'). ^{13}C NMR (126 MHz, d_2o) δ 175.2 (C-1') (C=O), 174.7 (C=O), 174.5 (C=O), 100.2 (C-2'), 97.2 (C-1), 70.3 (C-6'), 70.1 (C-4), 68.9 (C-4'), 68.4 (C-7'), 68.3 (C-5), 67.6 (C-3), 66.9 (C-8'), 65.5 (C-1''), 63.4 (C-6), 61.3 (C-9'), 52.2 (C-5'), 49.8 (C-2), 39.8 (C-3'), 37.3 (C-3''), 27.9 (C-2''), 22.1 (CH_3), 21.9 (CH_3). Found: $[M + H]^+$ 592.2324, $C_{22}H_{39}N_3O_{14}$ requires 592.2330.

Phenyl 4,6-*O*-benzylidene-2-deoxy-1-thio-2-(2,2,2-trichloroethoxycarbonyl-amino)-β-D-glucopyranoside (23). To a solution of thiophenyl 2-deoxy-2-(2,2,2-trichloroethoxycarbonylamino)-β-D-glucopyranoside [1] (429 mg, 0.96 mmol) and freshly distilled benzaldehyde dimethyl acetal (1.2 mmol) in CH_3CN (10 mL) was added $Cu(OTf)_2$ (17 mg, 0.048 mmol). The reaction mixture was sonicated at r.t. for 30 minutes under a nitrogen atmosphere until complete consumption of the starting material, as observed by TLC (CH_2Cl_2/MeOH, 9:1, v/v). The reaction mixture was quenched with Et_3N (200 μL), concentrated under reduced pressure and purified on silica gel flash colum chromatography to give product **23** (430 mg, 84%) as a syrup. $[\alpha]_D^{21}$ −21.8 (*c* = 0.004, CH_2Cl_2); ^{1}H NMR (400 MHz, $CDCl_3$) δ_H 7.56 – 7.45 (m, 4H, Ph), 7.42 – 7.31 (m, 6H, Ph), 5.55 (s, 1H, PhCH), 5.26 (d, 1H, $J_{NH,2}$ = 8.0 Hz, NH), 4.92 (d, 1H, $J_{1,2}$ = 10.5 Hz, H-1), 4.83 (d, 1H, *J* = 12.0 Hz, C*H*HCCl$_3$, *Troc*), 4.72 (d, 1H, *J* = 12.0 Hz, CH*H*CCl$_3$, *Troc*), 4.38 (dd, 1H, $J_{6a,6b}$ = 10.3 Hz, $J_{5,6a}$ = 4.6 Hz, H-6a), 4.05 (*br*. s, 1H, H-3), 3.79 (t, 1H, $J_{4,5}$ = $J_{5,6b}$ = 10.3 Hz, H-6b) 3.56 - 3.46 (m, 3H, H-2, H-4, H-5), 2.94 (*br*. s, 1H, OH); ^{13}C NMR (125 MHz, $CDCl_3$) δ_C 154.3 (C=O, *Troc*), 136.9, 132.9, 129.4, 129.1, 128.4, 128.3, 126.3 (*aromatics*), 101.9 (Ph*C*H), 100.0 (*C*Cl$_3$, C_q), 86.6 (C-1), 81.2, 74.6 (CH_2, *Troc*), 72.3 (C-3), 70.3, 68.5 (C-6), 57.2; HRMS: (ESI$^+$) Found: $[M + Na]^+$ 556.0118, $C_{22}H_{22}Cl_3NaNO_6S^+$ requires 556.0126 (^{35}Cl).

Phenyl 2,3,4,6-tetra-*O*-acetyl-β-D-galactopyranosyl-(1→3)-*O*-4,6-*O*-benzylidene-2-deoxy-1-thio-2-(2,2,2-trichloroethoxycarbonyl-amino)-β-D-glucopyranoside (24). To a solution of glycosyl donor **13** (30 mg, 0.088 mmol) and acceptor **23** (30 mg, 0.052 mmol), 4 Å MS in CH_2Cl_2 (250 μL) was added TMSOTf (20 μL, 10% solution in CH_2Cl_2) at −78 °C. The reaction mixture was stirred for 3 h over which period it was allowed to warm to r.t. The reaction was quenched with Et_3N (20 μL) and purified by silica gel column chromatography (*n*-hexanes/EtOAc, 7:3, v/v) to afford disaccharide **24** (30 mg, 62%). $[\alpha]_D^{21}$ +26 (*c* = 0.002, H_2O); ^{1}H NMR (400 MHz, $CDCl_3$) δ_H 7.52 – 7.50 (m, 2H, Ph), 7.42 – 7.35 (m, 6H, Ph), 7.29 – 7.28 (m, 2H, Ph), 5.31 (d, 1H, $J_{NH,2}$ =10.0 Hz, NH) 5.30 (*br*. d, 1H, $J_{3',4'}$ = 3.5 Hz, H-4'), 5.10 (t, 1H, $J_{3,4}$ = $J_{2,3}$ = 9.5 Hz, H-3), 5.02 (dd, 1H, $J_{1',2'}$ = 8.0 Hz, $J_{2',3'}$ = 10.0, H-2') Hz, 4.85 (dd,

1H, $J_{2',3'}$ = 10.0 Hz, $J_{3',4'}$ = 3.5 Hz, H-3'), 4.79 (d, 1H, *J* = 12.0 Hz, C*__H__*HCCl$_3$), 4.75 (d, 1H, *J* = 12.0 Hz, CH*__H__*Cl$_3$), 4.73 (d, 1H, *J* = 12.0 Hz, C*__H__*HPh), 4.71 (d, 1H, $J_{1,2}$ = 10.0 Hz, H-1), 4.52 (d, 1H, *J* = 12.0 Hz, CH*__H__*Ph), 4.47 (d, 1H, $J_{1',2'}$ = 8.0 Hz, H-1'), 4.09 – 4.06 (m, 2H, H-6a and H-6b), 3.95 (t, 1H, $J_{3,4}$ = $J_{4,5}$ = 9.5 Hz, H-4), 3.82 – 3.65 (m, 4H, H-2, H-5' H-6a', H-6b'), 3.49 (dt, 1H, $J_{5,6a}$ = $J_{5,6b}$ = 2.5 Hz, $J_{4,5}$ = 9.5 Hz, H-5), 2.12 (s, 3H, CH_3), 2.06 (s, 3H, CH_3), 2.03 (s, 3H, CH_3), 1.97 (s, 3H, CH_3), 1.95 (s, 3H, CH_3); ^{13}C NMR (100 MHz, $CDCl_3$) δ_C 170.6, 170.3,170.2, 170.0.168.9 (5 × C=O, *Ac*), 154.1 (C=O, *Troc*), 137.7, 132.7 (2 × C_q, *aromatic*), 132.5, 128.9, 128.6, 128.1, 128.0, 127.9 (CH, *aromatics*), 100.4 (C-1'), 87.3 (C-1), 78.9 (C-5), 74.6 (C-4), 74.5 (CH_2, *Troc*), 73.7 (*__C__*H$_2$Ph), 73.5 (C-3), 70.9 (C-3'), 70.5 (C-5'), 69.1 (C-2'), 67.5 (C-6), 66.8 (C-4), 60.9 (C-6'), 55.1 (C-2), 20.8, 20.7, 20.6, 20.6 20.5 (5 × CH_3). HRMS: (ESI$^+$) Found: [M + Na]$^+$ 930.1323, $C_{38}H_{44}Cl_3NNaO_{16}S^+$ requires 930.1339 (^{35}Cl).

3-Azidopropyl 2,3,4,6-tetra-*O*-acetyl-β-D-galactopyranosyl-(1→3)-4,6-*O*-benzylidene-2-deoxy-2-(2,2,2-trichloroethoxycarbonylamino)-β-D-glucopyranoside-(1→3)-4,6-*O*-benzylidene-2-(2,2,2-trichloroethoxycarbonylamino)-α-D-galactopyranoside (25). To a solution of glycosyl donor **26** (60 mg, 0.07 mmol), glycoside acceptor **11** (28 mg, 0.05 mmol) and 4 Å MS in CH_2Cl_2 (0.8 mL) was added NIS (31 mg, 0.14) and TMSOTf (27 µL, 10% solution in CH_2Cl_2) at −40 °C. The reaction mixture was stirred for 4 h until complete consumption of the acceptor was observed by TLC. The reaction was quenched with Et_3N (50 µL) and purified by silica gel column chromatography (PhMe/EtOAc, 8:2 → 7:3, v/v) to afford trisaccharide **27** (53 mg, 78%). $[\alpha]_D^{19}$ +20 (*c* = 1.00, CH_2Cl_2); ^{1}H NMR (500 MHz, DMSO) δ_H 7.62 (d, *J* = 8.5 Hz, NH), 7.46-7.44 (m, 4H , Ph), 7.40-7.35 (m, 11H, Ph and NH), 5.64 (s, 1H, PhC*__H__*), 5.56 (s, 1H, PhC*__H__*), 5.22 (*br*. d, 1H, $J_{3',4'}$ = 3.5, H-4'), 5.12 (d, 1H, *J* = 12.0 Hz, C*__H__*HCCl$_3$), 5.02 (dd, 1H, $J_{3'',4''}$ = 3.5 Hz, $J_{2'',3''}$ = 10.5 Hz, H-3''), 4.91-4.85 (m, 3H, H-1, H-1', H-2''), 4.82 (d, 1H, $J_{1'',2''}$ = 8.0 Hz, H-1''), 4.78 (d, 1H, *J* = 12.0 Hz, C*__H__*HCCl$_3$), 4.72(d, 1H, *J* = 12.0 Hz, CH*__H__*CCl$_3$), 4.53 (d, 1H, *J* = 12.0 Hz, CH*__H__*CCl$_3$), 4.33 (*br*. d, 1H, $J_{3,4}$ = 3.0 Hz, H-4), 4.26 (dd, 1H, $J_{5'',6a''}$ = 4.5 Hz, Hz, $J_{6a'',6b''}$ = 10.0 Hz, H-6a''), 4.09-4.00 (m, 5H),

3.97 (dd, 1H, $J_{5,6a}$ = 7.0 Hz, $J_{6a,6b}$ = 11.0 Hz, H-6a), 3.92-3.88 (m, 2H, H-2', H-6b), 3.78 (t, $J_{5'',6b''}$ = $J_{6b'',6a''}$ = 10.0 Hz, H-6b''), 3.70-3.66 (m, 3H), 3.49-3.36 (m, 5H, H-2, H-5'', OC*H*H, $CH_2C\underline{H_2}N_3$), 2.05 (s, 3H, CH_3), 1.91 (s, 3H, CH_3), 1.89 (s, 3H, CH_3), 1.88 (s, 3H, CH_3), 1.82-1.78 (m, 2H, $OCH_2C\underline{H_2}$). ^{1}H NMR (125 MHz, DMSO) δ_C 169.8, 169.7, 164.4, 169.3 (4 × C=O, *Ac*), 154.4, 154.0 (2 × C=O, *Troc*), 138.5, 137.6 (2 × C_q, *aromatics*), 128.9, 128.2, 128.0, 127.8, 126.2, 126.0 (CH, *aromatics*), 101.8 (C-1'), 99.8, 99.6 (2 × Ph*C*H), 99.4 (C-1''), 97.3 (C-1), 96.3, 95.5 (2 × $\underline{C}Cl_3$ *Troc*), 77.9, 77.3, 74.8 (C-4''), 73.6, 73.5 (2 × CH_2, *Troc*), 73.0, 70.3 (C-3''), 69.5, 68.4 (C-2' and C-6'), 67.7 (C-6''), 67.1 (C-4''), 65.7, 64.5, 62.6, 60.8 (C-6), 57.1 (C-2'), 50.7, 47.8 ($\underline{C}H_2N_3$), 28.2 ($\underline{C}H_2CH_2N_3$), 20.4, 20.3, 20.3, 20.2 (4 × CH_3); HRMS: (ESI$^+$) Found: $[M + H]^+$ 1300.1493, $C_{49}H_{57}Cl_6N_5NaO_{22}$ requires 1300.1519 (^{35}Cl).

3-Azidopropyl β-D-galactopyranosyl-(1→3)-2-deoxy-2-acetamido-β-D-glucopyranoside-(1→3)-2-deoxy-2-acetamido-α-D-galactopyranoside, hydrochloride (7). To a solution of trisaccharide **25** (40 mg, 0.031 mmol) in MeOH (2 mL) *p*-TsOH monohydrate (1.2 mg, 0.006 mmol) was added. After sonicating for 30 min the reaction was quenched with Et_3N (50 μL) and the solvent was evaporated under reduced pressure. The residue was dissolved in THF (1 mL) and LiOH (1 mL, 1 M) was added. The reaction mixture was stirred at r.t. for 18 h until complete hydrolysis of acetate and trichlorocarbamate groups was observed by TLC (iPrOH/H_2O/NH_4OH, 7:3:1). After neutralising with HCl (0.1 M), the reaction mixture was co-evaporated with toluene under reduced pressure and the dried residue was re-dissolved in pyridine (2 mL) and Ac_2O (1 mL). The reaction mixture was allowed to stir at r.t. for a further 16 h until complete acetylation was observed by TLC (CH_2Cl_2/MeOH, 9:1). The mixture was concentrated under reduced pressure and the residue was dissolved in MeOH (2 mL) and sodium methoxide (0.2 mg, 3 μmol) was added and the reaction mixture was stirred at r.t. for 3 h until complete deacetylation as observed by TLC (iPrOH/H_2O/NH_4OH, 7:3:1). The reaction was neutralised with HCl (0.1 M) and the solvent evaporated under reduced pressure and then re-dissolved in 5% HCl in EtOH (2 mL). Pd/C (20 mg, 10 wt %) was added to the reaction mixture and it was placed

under an atmosphere of H_2 for 16 h. The reaction mixture was then filtered through Celite®. The filtrate was concentrated under reduced pressure and purified by C_{18} column chromatography (H_2O → H_2O/MeOH, 19:1, v/v) to yield the hydrochloride salt of trisaccharide **7** (11 mg, 54%) as white foam. $[\alpha]_D^{21}$ +26 (*c* = 0.002, H_2O); ^{1}H NMR (500 MHz, d_2o) δ = 4.67 (d, 1H, $J_{1,2}$=3.8, H-1), 4.44 (d, 1H, $J_{1'',2''}$=8.8, H-1''), 4.29 (d, 1H, $J_{1',2'}$=7.6, H-1'), 4.13 – 3.98 (m, 2H, H-2, H-4''), 3.87 – 3.23 (m, 16H, H-3, H-4, H-4', H-2'', 2 × H-6, H-2''', OC$\underline{H_2}$, H-6, 2 × H-6, H-3'', H-5'', H-5', H-5), 2.96 (t, 2H, *J*=6.8, C$\underline{H_2}$$NH_2$), 1.92 – 1.78 (m, 8H, CH3, CH3, C$\underline{H_2}$$CH_2NH_2$); ^{13}C NMR (126 MHz, d_2o) δ = 177.0 (C=O), 176.4 (C=O), 103.1(C-1'), 101.9 (C-1''), 97.0 (C-1), 82.1 (C-3'), 76.5 (C-3), 75.2 (C-3''), 75.0 (C-5), 72.5 (C-5''), 70.7 (C-4), 70.6 (C-5'), 70.4 (C-4'), 68.7 (C-4''), 68.5 (C-2'), 64.9 (C-6), 64.9 (OC$\underline{H_2}$), 61.1 (C-6), 60.3 (C-6), 54.6 (C-2''), 48.4 (C-2), 37.1 ($\underline{C}H_2NH_2$), 26.8 ($\underline{C}H_3NH_2$), 22.1 (CH_3), 22.3 (CH_3). Found: $[M + H]^+$ 644.2873, $C_{25}H_{46}N_3O_{16}$ requires 644.2878.

Key NMR signals of compounds **1** [2], **2** [3], **3** [2], **4** [3], **5** [3], and **6** [4] are consistent with the reported data for similar compounds.

References

1. Tran, A. T.; Jones, R. A.; Pastor, J.; Boisson, J.; Smith, N.; Galan, M. C. *Adv. Synth. Catal.* **2011,** *353,* 2593–2598. doi:10.1002/adsc.201100228
2. Kuduk, S.D.; Schwarz, J.B.; Chen, X.T.; Glunz, P.W.; Sames, D.; Ragupathi, G.; Livingston, P.O.; Danishefsky S.J. *J. Am. Chem. Soc.* **1998**, *120*, 12474-12485.
3. Mathieux, N.; Paulsen, H.; Meldal, M.; Bock, K. *J. Chem. Soc. Perkin. Trans. 1*, **1997**, 2359-2368.
4. Liebe, B.; Kunz, H. *Helv. Chim. Acta*, **1997**, *80*, 1473-1482.
